# Living on the Edge: The Goldilocks Zone of Polyomavirus Replication and Persistence

**DOI:** 10.3390/v18050571

**Published:** 2026-05-19

**Authors:** Wenqing Yuan, Sheila A. Haley, Michael J. Imperiale, Walter J. Atwood

**Affiliations:** 1 Department of Molecular Biology, Cell Biology, and Biochemistry, Brown University, Providence, RI 02912, USA; 2 Graduate Program in Pathobiology, Brown University, Providence, RI 02912, USA; 3 Department of Microbiology and Immunology, University of Michigan, Ann Arbor, MI 48109, USA

**Keywords:** polyomavirus, JCPyV, BKPyV, viral persistence, progressive multifocal leukoencephalopathy, polyomavirus nephropathy

## Abstract

BK and JC Polyomaviruses (BKPyV and JCPyV) are ubiquitous human pathogens capable of establishing lifelong, asymptomatic persistence in the majority of the global population. While decades of research have focused on their lytic replication cycles and the development of severe diseases, such as polyomavirus-associated nephropathy (PVAN) caused by BKPyV and progressive multifocal leukoencephalopathy (PML) caused by JCPyV, their primary evolutionary strategy is one of persistence rather than pathogenesis. This review shifts the perspective from a replication-centric framework towards an evolutionary persistence model, detailing the multi-layered host and viral determinants that maintain the homeostatic balance. At the cellular level, viral genomes are restricted by chromatinization into minichromosomes and host S-phase licensing. These constraints are reinforced by innate immune sensing and adaptive T-cell and antibody responses that curtail systemic dissemination while permitting periodic, low-level urinary shedding, which is essential for horizontal transmission. In addition to these host barriers, the viruses utilize intrinsic regulatory mechanisms to prevent excessive replication and immune detection, including the stable archetype non-coding control region (NCCR), viral microRNAs that downregulate early gene expression, and the small t antigen (STAg). Finally, we address unresolved questions regarding the full spectrum of cellular reservoirs, the molecular triggers of reactivation, and the ecological factors shaping their transmission routes. Understanding these maintenance mechanisms is crucial for refining clinical interventions and managing the rare, devastating transitions from silent persistence to lytic disease.

## 1. Introduction

Both BK and JC Polyomaviruses (BKPyV and JCPyV, respectively) are members of the genus *Betapolyomavirus* within the family Polyomaviridae, a classification based primarily on the phylogeny of the major early protein large T antigen (LTAg) and conserved genome organization. Within the genus *Betapolyomavirus*, BKPyV and JCPyV form a closely related clade of human-adapted viruses, sharing conserved regulatory architecture and a common strategy of lifelong persistence [1,2]. They are both non-enveloped icosahedral viruses [2,3,4], which were independently identified one after another in 1971 from patients with immunosuppression-associated diseases, marking the first recognition of human polyomaviruses [5,6]. The initial stages of infection for these viruses are dictated by specific host–cell surface interactions involving different receptors. BKPyV primarily utilizes α2,3-linked sialic acid-containing gangliosides, such as GD1b and GT1b, for attachment, whereas JCPyV specifically targets the sialic acid-containing pentasaccharide LSTc and requires the serotonin receptor 5HT2A as a critical entry co-receptor [7,8,9,10,11,12]. While BKPyV was isolated from a patient who had transplant-associated nephropathy [5], JCPyV was discovered from the brain tissue of a patient who had progressive multifocal leukoencephalopathy (PML) [6].

Both polyomavirus genomes consist of circular double-stranded DNA organized into early and late coding regions separated by non-coding control regions (NCCR), which govern transcription and DNA replication. While rearrangements of the NCCRs are frequently observed in disease-associated isolates, non-rearranged archetype configurations dominate in healthy individuals, suggesting that pathogenic variants do not reflect the default biological state of these viruses [13,14].

Despite the fact that their initial discovery was associated with pathology, subsequent epidemiological studies revealed that primary infection with both viruses is common, typically occurring in childhood, followed by lifelong persistence [15,16]. Both polyomaviruses are endemic viruses, with seroprevalence rates exceeding 70–90% in adult populations worldwide [4,16,17,18]. Additionally, primary infection with BKPyV and JCPyV is asymptomatic, with no clearly defined clinical syndrome associated with initial exposure [2,19]. The renal tubular epithelium and the urinary tract are the predominant sites where BKPyV and JCPyV persist [20,21]. Despite the absence of clinical symptoms, persistent infection is not completely silent. Numerous studies have shown that BKPyV is shed in the urine more commonly than JCPyV in immunocompromised individuals; however, among healthy individuals specifically, JCPyV shedding tends to be more commonly detected than BKPyV [17,22,23]. The paradox of differential shedding of JCPyV vs. BKPyV may be explained by more vigorous cellular and humoral immune responses to BKPyV [17]. Overall, urinary shedding likely represents a key mechanism by which BKPyV and JCPyV are maintained within human populations, allowing efficient horizontal transmission without compromising host survival. Some recent longitudinal studies using murine polyomavirus (MuPyV) have also shown that the shedding fate of the virus is established early after kidney infection and remains remarkably stable during persistence, with only a subset of kidney-resident viral lineages contributing to long-term urinary shedding, which reconciles widespread transmission with durable persistence through reservoir-associated reactivation [24,25]. From an evolutionary perspective, this pattern supports the idea that polyomaviruses have adapted to balance low-level replication sufficient for transmission with tight control mechanisms that prevent excessive viral amplification and host damage. In this review, we want to shift focus from replication strategies and rearranged variants to persistence as an evolved life strategy.

### 1.1. Replication of BK and JC Polyomaviruses

Decades of work have emphasized replication efficiency, viral variants, rearranged NCCR, and mechanisms of lytic infection in discussions of viral pathogenicity, as they are strongly associated with the diseased state. Central to this replication-centric framework has been the study of the NCCR, which contains the origin of replication as well as bidirectional promoters that regulate both early and late gene transcription [26]. In both BKPyV and JCPyV, rearranged NCCR configurations are frequently detected in clinical isolates derived from patients with nephropathy or PML [26,27,28,29]. These rearrangements often involve duplications, deletions, or reorganization of transcription factor binding sites, leading to increased early promoter activity and elevated LTAg expression [30,31]. Since LTAg drives viral DNA replication by recruiting host replication machinery [32], enhanced early transcription directly correlates with increased viral genome amplification and productive lytic infection. Both in vivo and in vitro studies have demonstrated that rearranged NCCRs exhibit higher replication capacity compared to archetypal forms, strengthening the idea that NCCR plasticity is a major determinant of pathogenic potential [13,27,28].

In addition to NCCR rearrangements, other viral and host factors that contribute to lytic replication have been investigated. For instance, LTAg can enhance replication efficiency by forcing cells into G2 arrest [33], while the small t antigen (STAg) exhibits slightly different functions in BKPyV and JCPyV. Though STAg in both viruses is found to modulate signaling pathways through PP2A, the STAg of JCPyV is essential for efficient viral replication [34], whereas the STAg of the BKPyV virus acts in the opposite direction, inhibiting LTAg expression and viral DNA replication [35]. Additionally, in contrast to BKPyV, VP2 and VP3 minor capsid proteins of JCPyV are found to actively contribute to viral DNA replication by interacting with Hsp70 [36]. Host determinants that enhance replication have also been characterized, including cellular transcription factors, such as the NFAT family, which are active in glial cells during JCPyV infection or in renal epithelial cells during BKPyV infection [37,38]. Notably, SP1 has also been shown to bind the NCCR and promote early gene transcription of BKPyV, which influences viral reactivation and replication dynamics in human renal epithelial cells in vitro [39]. Overall, previous work has provided a detailed understanding of how polyomaviruses amplify their genomes, hijack cellular machinery, and ultimately induce cytolytic infection in permissive tissues.

### 1.2. Persistence of BK and JC Polyomaviruses

Traditional research paradigms have focused on viral replication efficiency and pathogenicity, often emphasizing rearranged NCCR variants due to their association with disease. However, from an evolutionary perspective, BKPyV and JCPyV appear optimized for persistence rather than acute replication. Both BKPyV and JCPyV establish persistent infections following asymptomatic primary exposure, most often during childhood. Viral genomes can be detected in tonsil or renal tissues of healthy individuals [40,41], while intermittent viruria occurs in a significant proportion of immunocompetent adults [42,43]. Viral genomes exist as chromatinized minichromosomes associated with host histones, subject to cellular epigenetic control [44,45]. This chromatinization likely restricts transcriptional output under homeostatic conditions, enabling long-term maintenance without triggering robust immune activation. In addition, a defining feature of polyomavirus biology in immunocompetent hosts is the absence of clinically apparent inflammation during persistent infection. Several viral mechanisms appear to contribute to this immune-compatible state, including LTAg downregulation by viral microRNA and reduced transcription with the archetype NCCR [46,47,48]. From an evolutionary standpoint, lifelong persistence maximizes opportunities for transmission over the host’s lifespan, making aggressive lytic replication unnecessary. Therefore, polyomaviruses are not “designed” to cause disease; instead, they evolved to establish lifelong persistence by replicating periodically at low levels to ensure transmission and minimizing host damage.

For both BKPyV and JCPyV, disease emerges only when the host–virus equilibrium is disrupted by profound immunosuppression. However, the scenarios for each virus occur slightly differently. Immunosuppression, particularly in advanced HIV/AIDS, is strongly associated with JCPyV reactivation and the development of PML [44], and, more recently, JCPyV-associated PML has also been increasingly recognized in HIV-uninfected individuals rendered immunosuppressed by medical therapies, including biologics/monoclonal antibodies used for autoimmune disease and cancer [43,49]. This shift was first seen when PML was confirmed in 2005–2006 in patients treated with natalizumab for multiple sclerosis and Crohn’s disease, conditions not previously associated with PML [50]. Additionally, iatrogenic immunosuppression during solid organ or hematopoietic stem cell transplantation frequently leads to BKPyV reactivation and polyomavirus-associated nephropathy and hemorrhagic cystitis, respectively [51]. Acquisition of immunosuppression or immunosuppressive therapy often leads to the failure of viral control, further supporting the central role of immune balance in determining disease outcome.

## 2. Cellular Determinants of Persistence

### 2.1. Cellular of BK and JC Polyomaviruses

**Figure 1:**
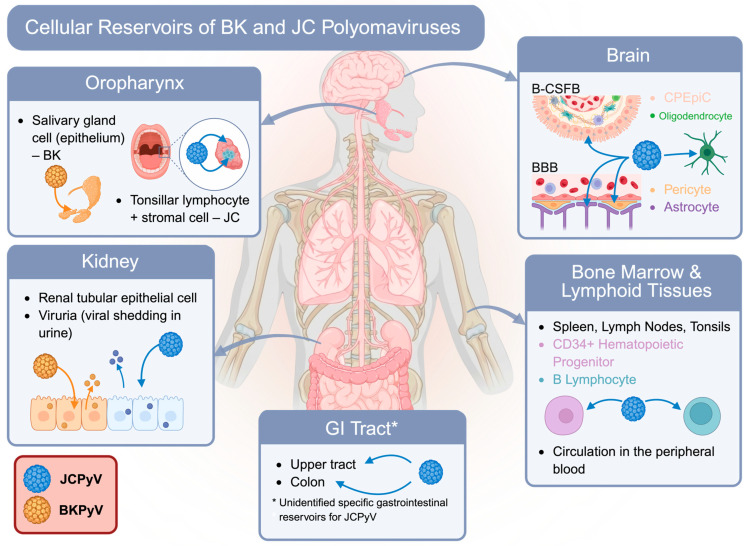
Cellular reservoirs of BK and JC polyomaviruses. *A schematic summary of established and proposed tissue sites that support persistent infection of BKPyV and JCPyV. Both viruses establish latency in the kidney, particularly in renal tubular epithelial cells, where viruria is commonly observed in both healthy and immunocompromised individuals. The oropharynx is also a potential site of early infection or persistence, including salivary gland epithelium for BKPyV and tonsillar lymphocytes and/or stromal cells for JCPyV. In contrast to BKPyV, JCPyV exhibits a broader tissue distribution, with detection in bone marrow and lymphoid organs, including spleen, lymph nodes, tonsils, circulating CD34^+^ hematopoietic progenitors, and B lymphocytes, supporting its potential for peripheral dissemination by immune-cell trafficking. JCPyV has also been identified in both the upper and lower GI tract, though the precise cellular niches for the virus within the gastrointestinal system have yet to be characterized. Upon reactivation, JCPyV acquires neurotropism by invading both the blood–brain barrier (BBB) and blood-cerebrospinal fluid barrier (B-CSFB), thereby rendering brain cells, including choroid plexus epithelial cells (CPEpiCs), brain vascular pericytes, and oligodendrocytes, which contribute to the development of PML; whereas BKPyV reactivation is primarily linked to kidney-centered disease, including BK virus-associated nephropathy (BKVAN). Created with Biorender.com*.

The kidney represents the most robustly supported site of persistent infection for both BKPyV and JCPyV. Viral DNA, which persists in renal tubular epithelial cells, is frequently detected in urine from healthy individuals, and asymptomatic viruria is common [52,53,54]. Studies have detected BKPyV in kidney tissues and urine of immunocompetent individuals, indicating that the renal epithelium harbors a subclinical infection that can reactivate under immunosuppression [54,55]. Similarly, JCPyV is known to remain quiescent in the kidneys, contributing to intermittent viral shedding in urine, which has been observed in about 20% of healthy individuals without clinical symptoms [47,56]. Unlike BKPyV, which is largely confined within the urinary tract, JCPyV has been detected in several extra-renal sites, such as in bone marrow, upper and lower gastrointestinal tract, and lymphoid tissues, including spleen, lymph nodes, and tonsils of healthy or immunosuppressed individuals [40,56,57,58,59,60,61]. JCPyV is also reported to infect circulating B lymphocytes and CD34+ hematopoietic progenitor cells, as demonstrated by cell culture followed by PCR [57], leading to the proposal that JCPyV might latently persist in immune cells and use them as carriers during reactivation (Figure 1). There is one study that found the viral DNA of JCPyV in normal brain tissue from patients without PML [56], raising an intriguing question of whether JCPyV could actually establish a low-level persistent infection in glial cells or precursors in the central nervous system. However, other autopsy studies failed to identify the viral genome in normal brain tissue, an inconsistency that remains unexplained and suggests that the central nervous system or bone marrow latency of JCPyV remains uncertain [62].

Another key distinction of JCPyV is the possibility of cell-type switching during reactivation (Figure 1). The virus appears to undergo genetic and tropism changes when transitioning from latency to productive infection. In healthy carriers, archetype JCPyV containing a stable NCCR has limited replication in the kidney. Upon immunosuppression, JCPyV undergoes reactivation and rearrangement of NCCR, which not only enhances its replication but also exhibits novel glial cell tropism, infecting oligodendrocytes and astrocytes in the brain, compared to the archetype [4,14,56]. The development of PML is linked to the emergence of these neurotropic JCPyV variants that destroy myelin-producing oligodendrocytes in the CNS [4,63,64]. JCPyV undergoes this adaptive switch in cell tropism, possibly prior to or during migration to the brain, moving from a latent infection in renal or lymphoid cells to a lytic infection in glial cells after the viral genome rearranges to form more aggressive variants. The change in tissue specificity of JCPyV due to reactivation, leading to neuropathology, is in marked contrast to BKPyV, which generally reactivates in its original reservoir, the kidney, and causes localized disease (e.g., BK-associated nephropathy). However, in studies conducted in primary cells, BKPyV-infected microvascular endothelial cells (lung and bladder) exhibited a persistent/low-yield infection phenotype (prolonged viral protein expression, delayed cell death) that contrasted with the more lytic outcome observed in renal proximal tubule epithelial cells. This cell-type–dependent phenotype supports the possibility that BKPyV can also “shift” the cellular compartment it occupies during spread or reactivation, even if most recognized BKPyV pathology remains centered on the kidney and urinary tract [65].

### 2.2. Restrictions of Polyomaviruses by Cellular Factors

**Figure 2:**
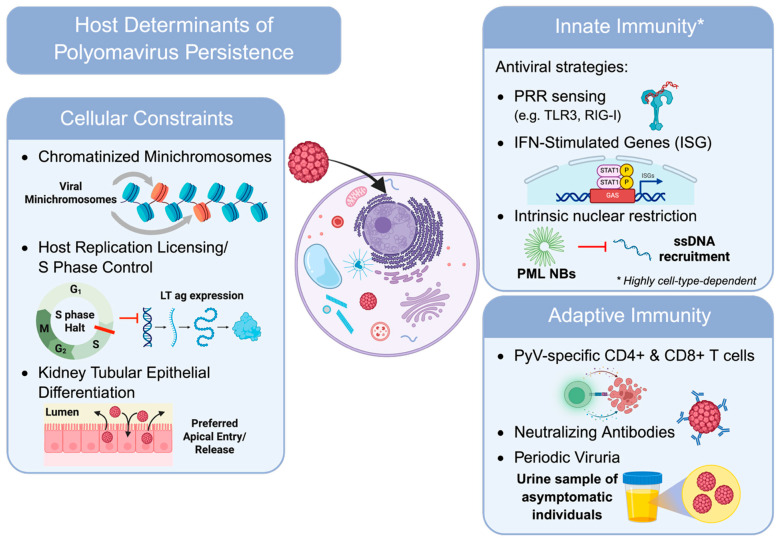
Host determinants that constrain BKPyV and JCPyV persistence. *Selected host mechanisms that limit productive replication and favor the persistence of human polyomaviruses are shown. At the cellular level, viral genomes are maintained as chromatinized minichromosomes, replication remains dependent on host licensing and S-phase competence, and the quiescent, differentiated state of renal tubular epithelium imposes an additional barrier to lytic amplification. Innate immune control is represented by PRR signaling, IFN-stimulated effector programs, and intrinsic nuclear restriction pathways, which together can suppress early viral gene expression and replication in a strongly cell-type-dependent manner. Adaptive control is illustrated by virus-specific T-cell responses, neutralizing antibodies, and the interpretation of periodic asymptomatic viruria as a marker of dynamic but contained host–virus equilibrium rather than complete virologic silence/elimination. Created with Biorender.com*.

The key point about BKPyV and JCPyV persistence is that they do not replicate robustly most of the time. There are three cellular bottlenecks regulating persistence: (i) viral genomes exist as chromatinized minichromosomes to suppress early transcription, (ii) the control of host replication licensing and S phase factor to constrain genome amplification, and (iii) the differentiation and cycling of kidney tubule epithelium as a gatekeeper for lytic replication (Figure 2).

Several DNA viruses, including BKPyV and JCPyV, upon entering the host cell nucleus, have their viral DNA chromatinized and associated with host histone proteins to form structures resembling cellular chromatin, commonly known as chromatinized minichromosomes [66,67]. For BKPyV, its virion DNA can be extracted as a nucleoprotein complex containing around 20 nucleosomes, which can reduce both initiation and elongation of in vitro transcription relative to purified superhelical DNA [68]. Recently, another study using virus-specific molecular profiling determined that NCCR-rearranged BKPyV minichromosomes carry a diverse array of histone post-translational modifications, including hyperacetylation, which is often associated with transcriptional competence [69]. Instead of implying a uniform repression, this finding actually demonstrated that BKPyV chromatin is dynamically regulated and capable of rapid switching between permissive and restrictive states depending on the cellular context. For JCPyV, the histone acetylation state is highly cell-type-specific in terms of early promoter accessibility. The acetylation of JCPyV early promoter is found in glial cells but not in nonglial cells (HeLa), and histone deacetylase (HDAC) inhibition is able to induce promoter hyperacetylation in HeLa, while HDAC inhibitors like sodium butyrate can activate the JCPyV promoter ~20–30-fold in nonglial cells but have minimal effect in glial cells [69]. In such a scenario, chromatinization helps enforce restricted early transcription, thereby restricting entry into the lytic cycle (Figure 2).

Polyomaviruses rely heavily on host DNA replication machinery, making cell-cycle positioning and licensing capacity decisive for genome amplification. For BKPyV, it was found in primary human renal proximal tubule epithelial cells that robust LTAg expression and viral production depended on an initial host S phase, meaning that either inhibition of host origin licensing or origin firing before re-replication or the canonical origin-firing kinases Cdc7 and Cdk2 would decrease viral titers and prevent robust LTAg expression [70]. Another host restriction study has shown that purified BKPyV LTAg supported origin-dependent replication with human but not murine extracts in vitro, and murine extracts inhibited BKPyV LT–dependent replication at a step prior to or during origin unwinding, and was not rescued by adding several human replication proteins [71], which suggests that even with viral helicase present, certain cellular replication contexts may block origin activation. For JCPyV, cell-cycle manipulation appears in different phases. In infected and LTAg-transfected systems, JCPyV LTAg induced ATM/ATR-mediated G2 checkpoint signaling and G2-arrest accumulation, which promoted viral replication [33] (Figure 2). The likely mechanism behind this is that JCPyV can promote a prolonged replication-competent state while suppressing mitotic completion, thereby increasing access to replication/repair factors and reducing competition with host genome duplication programs. The scenarios for both viruses align with the broader concept that a low-level state of persistence may be maintained partly by cells that are replication-incompetent or actively suppressing origin firing.

As the best-supported reservoir for both BKPyV and JCPyV, the kidney is predominantly composed of specialized epithelial cells that are normally slow-cycling or quiescent [72]. For BKPyV, if lytic replication requires S-phase licensing and firing, then a low-turnover epithelium would enforce a persistence-compatible bottleneck, permitting only sporadic replication in the minority of cells that transiently re-enter the cell cycle (e.g., during repair, inflammation, or local stress) [70]. Another study showed that cell culture drives a stressed/injured-kidney-like gene expression state in which BKPyV replicates preferentially during nephropathy [65,70]. A clinical observation has shown that productive BKPyV infection is coupled with cell-cycle perturbation in vivo, where early-phase infection (TAg+) was strongly associated with nuclear p53 accumulation and activation of the proliferation marker Ki67 in infected tubular cells of renal allografts with polyomavirus-associated nephropathy (PVAN) [73]. Additionally, epithelial differentiation includes polarity, which can affect entry, compartmentalized release, and potentially the local inflammatory visibility of replication. A polarized renal epithelial cell model showed preferential apical entry and apical release of progeny and infected cells with barrier maintenance early, proposing that low-level replication could disseminate through the tubular lumen while delaying immune detection [74].

Taken together, these constraints plausibly favor archetype-like, low-output regulatory programs compatible with persistence, while providing selective opportunity for rearranged control regions only under unusual tissue environments or immunosuppression. Evolutionarily, this plausibly selects for regulatory architectures (including archetype NCCR configurations) that can maintain extremely low basal transcription/replication in quiescent epithelia yet remain poised for limited, periodic amplification when host physiology transiently supplies S-phase competencies (repair, local inflammation, hormonal cues) or when immunosuppression reduces the cost of replication [70].

### 2.3. Restrictions of Polyomaviruses by Innate Immunity

Innate immunity constrains BKPyV and JCPyV at two overlapping levels: (i) pattern-recognition receptor (PRR) sensing that results in IFN-stimulated gene (ISG) effector programs that impose antiviral pressure, and (ii) intrinsic nuclear restriction systems that can gate early gene expression and amplify IFN-linked repression (Figure 2).

These restrictions are highly cell-type-dependent, where BKPyV can be “invisible” to antiviral programs in renal tubular epithelial cells yet strongly IFN-responsive in microvascular endothelial cells [65], while JCPyV is often IFN-sensitive in renal epithelial and glial systems [75]. Consistent with this “invisibility,” unbiased RNA-expression profiling in renal tubular epithelial infection has repeatedly found little to no induction of canonical IFN/innate immune transcriptional programs during BKPyV infection, and temporal host-proteome profiling likewise reports a striking lack of innate immune response throughout the BKPyV replication cycle [76]. Though infection by both viruses can induce interferon production, RNA-seq-based comparisons show that ISGs are robustly activated primarily in the JCPyV-infected cells and correlate with nuclear colocalization/translocation of phosphorylated STAT1 and IRF9, whereas BKPyV shows minimal ISG induction and instead induces SOCS3 and SOCS1 while downregulating components of the IFN signaling cascade [75,76]. Blockade of interferon (IFN-α and IFN-β) activity also partially weakens the blockade of JCPyV infection, indicating that a type I IFN response is essential for establishing a persistent but contained infection [75]. In JCPyV-infected glial cells, IFN-α/IFN-β has been shown to exert direct antiviral effects throughout the infectious cycle [77,78]. In addition, JCPyV infection of primary human choroid plexus cells induces the secretion of the chemokines CCL, CXCL5, CXCL6, and CXCL8 [79]. BKPyV sensing is more heterogeneous. In BKPyV-associated nephropathy, renal tissues exhibit upregulation of PRRs such as TLR3 and RIG-I, which sense viral or dsRNA intermediates and activate IRF3/IRF7-dependent signaling pathways, leading to induction of IFN-β and downstream antiviral ISGs in collecting duct epithelial cells, which establishes an antiviral state that constrains early viral gene expression and genome amplification [80] (Figure 2). In primary human microvascular endothelial cells, BKPyV infection results in IRF3 and STAT1 activation, IFN-β production, CXCL10 secretion, and broad ISG induction, which concurrently support a long-lived, low-yield infection compatible with persistence [65]. Mechanistically, BKPyV agnoprotein disrupts mitochondrial networks and membrane potential during late replication, impairing nuclear IRF3 translocation and IFN-β expression and promoting mitophagy—providing a direct route to innate immune blockade in otherwise sensing-competent cells [81]. Complementing these evasion strategies, JCPyV has been found to employ its own early protein to dampen sensing. Specifically, JCPyV STAg acts as an IFN antagonist by indirectly suppressing RIG-I-mediated signal transduction through interacting with the E3 ubiquitin ligase TRIM25 to induce the K63-linked ubiquitination of RIG-I [82]. Moreover, early gene loci from multiple polyomaviruses, including BKPyV, repressed TLR9 promoter activity and reduced TLR9 mRNA in some cellular contexts, implying a potential immune-evasion lever at the level of endosomal DNA sensing [83].

Once IFN-I signaling is engaged, ISG effector proteins impose diverse pressures on polyomaviruses, including direct inhibition of viral gene expression and “genome editing” that can accelerate within-host diversification. In terms of BKPyV, an ISG called MxA, induced by IFN-α, has been found to inhibit BKPyV replication by interacting with the BKPyV LTAg [84]. Additionally, an emerging class of ISG, ISG20, has been shown to act as a novel host restriction factor against BKPyV, while BKPyV employs LT-mediated mechanisms to evade or counteract ISG20’s antiviral effects [85]. In addition, expression of APOBEC3 cytidine deaminases was found to be consistent with mutational patterns by sequencing of BKPyV variants from kidney transplant recipients, suggesting APOBEC3 as a mutational force in vivo [86]. Beyond viral diversification, this mutational capacity may drive significant pathology, which has been shown in recent work that virus-induced APOBEC3 transmutagenesis is a causative association between polyomavirus persistence and bladder cancer during cancer initiation [87]. These findings suggest that deciphering the mechanisms of polyomavirus persistence may clinically impact a much larger population than previously thought, as either a chronic hyperactive innate immune response or an insufficient adaptive immune response may promote the persistent infection necessary to drive such oncogenic processes. For JCPyV, though there is no solid evidence to support APOBEC3-driven hypermutation, broad comparative analyses across human viruses identify polyomaviruses among the most footprinted families by APOBEC3-associated motif depletion, consistent with long-term selection pressure [88].

Intrinsic immunity differs from inducible IFN programs in that it is often constitutive, nuclear, and immediate, shaping whether incoming viral genomes can initiate early transcription. For small DNA viruses, the PML nuclear body (PML-NB/ND10), a key role player, can mediate intrinsic repression of viral replication through epigenetic silencing or genome entrapment, while also acting as nodes that reinforce innate/IFN responses [89] (Figure 2). Recent evidence has expanded our understanding of this virus–host standoff with the discovery of a novel JCPyV protein, ORF4, which specifically targets PML-NB complexes and alters their distribution within the nucleus [90]. This discovery is significant because, for JCPyV, modulation of nuclear PML-NB/ND10 levels inversely regulates viral infection, with PML-NB/ND10 depletion enhancing infection and PML enrichment restricting it [91,92,93]. Specifically, IFN-β-driven host response likely increases PML-NB/ND10 levels to reinforce intrinsic antiviral defenses against viral infection [91]. While it was previously thought that JCPyV infection could not directly modulate nuclear levels of PML-NB/ND10, the identification of ORF4 suggests a targeted viral strategy to reorganize these architectural defenses. For BKPyV, PML-NB/ND10 acts differently in comparison to their role in JCPyV infection, where they can accumulate newly synthesized viral DNA during viral replication, and PML-NB/ND10 suppression prevents recruitment of ssDNA into nuclear foci without significantly reducing overall viral DNA replication, suggesting that PML-NB/ND10 may function as scaffolds for DNA processing rather than a direct blockade of viral infection [94].

An evolutionary interpretation for all mechanisms above is that innate restriction acts as a selector of persistence phenotypes, where it not only blocks replication but also selects which replication programs are viable in vivo. Another way to interpret these is that the capacity of innate immune and intrinsic nuclear restriction likely defines an allowed space for persistent infection (favoring low-level replication programs such as archetype-like NCCRs and/or effective immune evasion), while immunosuppression expands this space and permits selection of high-expression NCCRs that would otherwise be counterselected by IFN/ISG activation and intrinsic repression.

### 2.4. Restrictions and Long-Term Control of Polyomaviruses by Adaptive Immunity

Adaptive immunity is essential to ensure that the virus does not win the battle with the host: (i) polyomavirus-specific CD8+ and CD4+ T cells limit productive viral replication [95], (ii) antibody responses reducing systemic dissemination and modulating viral kinetics after transplantation, and (iii) periodic, asymptomatic viruria reducing viral loads in healthy individuals [19] (Figure 2).

In kidney transplantation, BKPyV control is closely linked to the recovery and expansion of virus-specific T cell responses, with increased LTAg- and VP1-specific responses observed in infected renal epithelial compartments as viremia resolves [96]. In another cohort study, the presence of BKPyV-specific 9mer CD8+ T cell responses correlated with clearance of BK viremia, supporting the view that CD8-driven control is a key determinant of viral set point under immunosuppression [95]. For JCPyV, effective cellular immunity can re-establish control even after CNS invasion. A clinical immunologic study has shown that detectable JCPyV-specific CD8+ T cell responses are associated with improved outcome in PML patients [97]. Natalizumab-associated PML cases often exhibit absent or functionally skewed JCPyV-specific responses, like IL-10–dominant CD4 signatures, compared with non-PML controls [98] (Figure 2).

Neutralizing antibody (nAb) responses against BKPyV are genotype/serotype-specific, where there is limited cross-neutralization between major BK serotypes, which acts as an important constraint on protective breadth [99]. Before transplantation, the mismatched patterns of donor/recipient BKPyV seroreactivity and nAb can predict post-transplant BK DNAemia risk in large clinical cohorts, supporting nAbs as contributors to containment [100]. However, BKPyV nephropathy can occur despite substantial anti-BKPyV antibody levels, indicating that humoral immunity alone may be insufficient once high-level intrarenal replication is established [99]. For JCPyV, most adults do have circulating antibodies, yet these do not reliably prevent PML [101]. Additionally, PML-associated VP1 variants can evade neutralization relative to archetype wild-type strains, providing a concrete route by which humoral pressure may be bypassed in the diseased state [102].

Viruria of BKPyV and JCPyV is a frequent asymptomatic situation shown by population studies, with JCPyV excretion often more continuous and BKPyV more intermittent, yet this is evidence of equilibrium, not failure of infection control [19]. BKPyV viruria also varies with age in immunocompetent cohorts, consistent with episodic reactivation controlled by immune memory rather than unchecked expansion [103]. A plausible interpretation is that, in immunocompetent hosts, these episodes reflect intermittent, stochastic reactivation that is rapidly contained by adaptive immune memory, thereby preventing disease and re-establishing viral persistence. Specifically, JCPyV is commonly detected in urine but rare in blood, suggesting that systemic restriction, likely involving antibodies and T cells, is typically effective outside special immunosuppressed states [104].

## 3. Viral Determinants of Persistence

### 3.1. NCCR as the Evolutionary Strategy of Persistence

**Figure 3:**
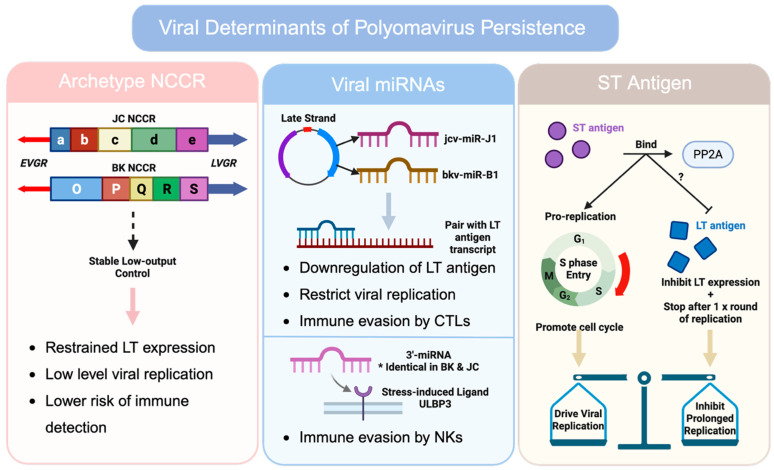
Viral determinants that favor BKPyV and JCPyV persistence. *Selected viral mechanisms are shown that bias polyomavirus infection toward persistent, low-output states. Archetype NCCR configurations are depicted as regulatory architectures associated with restrained early gene expression, limited replication, and reduced immune visibility relative to rearranged disease-associated variants. Viral microRNAs derived from the late strand are negative-feedback regulators of LTAg expression, thereby restricting replication while also reducing immune recognition; the shared 3′ miRNA effect on ULBP3 provides potential escape from NK cell surveillance. STAg is a context-dependent regulator that both promotes replication via PP2A-linked signaling and, particularly in BKPyV, can restrain excessive LT-driven amplification, consistent with a role in maintaining a replication balance compatible with persistence. Created with Biorender.com*.

Archetype NCCR structures predominate in the general asymptomatic population. Archetype JCPyV sequences are consistently detected in urine samples from healthy individuals, and similar patterns are observed for BKPyV [53,105]. The archetype configuration typically exhibits lower basal early promoter activity compared with rearranged forms, resulting in more restrained LTAg expression and reduced replication potential in vitro. Low-level replication also carries a lower risk of immune detection and causes less host tissue damage due to lytic replication. Rearranged NCCRs are frequently isolated from diseased tissues: renal allografts in BKPyV–associated nephropathy and brain tissue or cerebrospinal fluid in PML. In the diseased state, high-level replication is often accompanied by the emergence of rearranged NCCR variants that enhance early gene transcription (e.g., LTAg expression) and replication efficiency by increasing early promoter activity, as shown by functional studies [39,105,106] (Figure 3). Importantly, longitudinal analyses in transplant recipients suggest that NCCR rearrangements may arise during periods of intense replication under immunosuppression rather than being transmitted as dominant circulating forms or maintained in stable transmission chains [29]. Rearranged NCCRs are rarely detected in the absence of immunosuppression and appear to arise de novo or expand within individual hosts under altered selective pressures. In this sense, archetype NCCRs may represent evolutionarily stable strategies maintained by purifying selection across human populations.

### 3.2. Viral miRNA-Mediated Control of Early Gene Expression

Polyomaviruses, including BKPyV and JCPyV, encode their own microRNAs (miRNAs) that play a key role in long-term persistence. These viral miRNAs, bkv-miR-B1 in BKPyV and jcv-miR-J1 in JCPyV, are transcribed from the late strand of the viral genome and are complementary to early mRNAs encoding the LTAg [2,107,108]. The miRNA can pair with the LTAg transcript to trigger the downregulation of LTAg in infected cells, which serves as a negative feedback loop limiting LTAg expression, thus restricting viral replication during the asymptomatic persistent phase of infection [109]. Additionally, the excision of JCPyV miRNA substantially increased LTAg expression and restored viral replication in vitro [109], proving that viral miRNAs act as a brake on the lytic cycle. For BKPyV, however, this inhibitory effect appears to be most effective in the context of an archetype NCCR, suggesting that LTAg expression driven by rearranged NCCRs may exceed the suppressive capacity of the viral miRNA [47,48] (Figure 3).

In addition to suppressing viral replication, miRNAs are essential for immune evasion, where they can reduce the abundance of viral proteins that could be presented as antigens. BKPyV- and JCPyV-infected cells have low LTAg levels maintained by viral miRNAs so that they display few antigens on MHC class I, thereby diminishing recognition and killing by cytotoxic T lymphocytes [107,110]. Beyond LTAg, the viral miRNAs can target host immunity molecules. Specifically, an identical 3′-miRNA from JCPyV and BKPyV targets the stress-induced ligand ULBP3 on infected cells to help them escape killing by natural killer (NK) cells [111]. When ULBP3 is restored by blocking miRNA, it significantly increases NK cell–mediated lysis of infected cells [111]. Viral miRNAs facilitate persistent infection through both direct reduction in immunogenic viral proteins and targeting of host immune ligands (Figure 3).

Another importance of viral miRNAs in persistence is that loss or dysregulation of miRNA control may contribute to disease-associated replication. In the state of immunosuppression with low immune pressure, rare viral variants arise that carry mutations deleting or disrupting the miRNA genes [112]. In JCPyV, miRNA-attenuated or null variants identified in immunocompromised hosts can carry small insertions or deletions that abolish miRNA expression while preserving the amino-acid reading frame of the opposing-strand LTAg gene [109,112]. Because the miRNA precursor maps antisense to (and within) the LTAg coding exon, these indels can simultaneously disrupt miRNA production and introduce in-frame LTAg sequence changes, so the resulting “unabated LTAg” phenotype cannot be attributed unambiguously to miRNA loss versus mutant LTAg function [113]. In other typical PML cases, viral miRNA remains detectable at high levels in JCPyV-infected brain cells [109], suggesting the virus actively attempts to limit its own replication. Similarly, in biopsy tissues and plasma from patients with PVAN, BKPyV miR-B1 is expressed at high levels to suppress early gene expression [114], indicating that the miRNA response, although present, is insufficient to prevent tissue damage. To summarize, altered miRNA-mediated negative feedback, potentially together with linked sequence changes in the overlapping LTAg coding region [113], can be associated with elevated LTAg expression and high-level replication in disease settings, which are PML in the CNS for JCPyV and PVAN in the kidney for BKPyV.

### 3.3. ST Antigen as a DNA Replication Limiter

The STAg, a multifunctional viral regulator, contributes to an ideal balance of viral replication: enough to persist but not enough to kill the host. First, the STAg modulates the host serine/threonine phosphatase PP2A to modulate cell signaling pathways involved in replication control [2,115,116]. The STAg is able to indirectly drive cell cycle progression by binding to the PP2A scaffolding subunit to perturb its tumor suppression function [115]. This activity creates a cellular environment favorable for DNA synthesis. For JCPyV, the STAg similarly acts as a pro-replication factor, where it binds PP2A, which is required for efficient viral replication [34]. These findings underscore that STAg normally boosts polyomavirus replication by subverting host signaling checkpoints and promoting S-phase entry (Figure 3).

Surprisingly, recent studies have shown that the STAg can also function as a brake to prevent excessive replication in certain contexts. In BKPyV, the STAg indeed inhibits LTAg expression and viral DNA replication in the archetypal virus background [35]. Deleting the STAg gene from BKPyV increased early gene (i.e., LTAg) expression and genome copy number, whereas the presence of wild-type STAg curbed replication levels, partially dependent on STAg’s interaction with PP2A [35]. Consistent with this, JCPyV is also found to rely on its STAg for controlled maintenance of the viral genome. Absence of the STAg allows an initial round of DNA synthesis but cannot sustain prolonged replication, indicating that STAg is required to maintain viral DNA at low yet steady levels [2,34,116]. Overall, the STAg also serves a counterbalancing function to avoid uncontrolled viral replication that could harm the host cell (Figure 3).

The dual role of STAg suggests an evolutionary adaptation to maintain the virus within an optimized range of replication that is tolerable by the host. On one hand, the prototypical archetype strains of BKPyV and JCPyV (predominant in healthy carriers) have constrained replication profiles governed by their non-coding control regions and regulatory proteins like STAg. On the other hand, the involvement of STAg in dampening excessive genome replication by antagonizing PP2A and modulating growth signaling helps strike a balance between viral persistence and pathogenesis. In essence, STAg fine-tunes polyomavirus replication for long-term persistence, ensuring sufficient viral maintenance without tipping into host-damaging, immunogenic levels of replication.

## 4. Unresolved Questions and Future Directions

With decades of research on polyomaviruses, we now understand that BKPyV and JCPyV are highly prevalent human pathogens that establish persistent, mostly asymptomatic infections in healthy individuals, relying on a finely tuned balance between viral regulation and host immune control. While substantial progress has been made in characterizing their replication, transcriptional control, and pathogenesis during immunosuppression, critical aspects of their biology remain poorly understood. Chief among these are the true cellular reservoirs that support lifelong persistence, the molecular mechanisms that maintain latency or trigger reactivation, and the full spectrum of transmission routes and ecological factors shaping their life cycle. Addressing these gaps is essential not only for refining our understanding of polyomavirus–host coevolution but also for anticipating and mitigating disease emergence in vulnerable populations. As almost all the experimental work on BKPyV has been performed in the context of the nephropathy model (e.g., PVAN), we have not discussed hemorrhagic cystitis (HC) in any detail. We note, however, that the types of mechanisms we describe for PVAN may also be applicable to HC.

### 4.1. Unresolved Cellular Reservoirs

The kidney is well known to serve as a reservoir for both viruses, but it is unclear whether the kidney is the exclusive persistent reservoir. For BKPyV, lifelong latency is usually established in renal epithelial cells, especially the renal tubules, and it may also infect urothelial cells in the ureters and bladder [117]. These cells provide a reservoir where BKPyV can persist asymptomatically, owing to minimal immune surveillance in the kidney. Recently, glomerular cells have been shown to be susceptible to BKPyV infection [55], while rare involvement of BKPyV in glomerular tissue has been observed in vivo. However, such findings hint that persistent infection may not be strictly confined to a single epithelial lineage. The association of BKPyV with hemorrhagic cystitis also raises the possibility that the virus can persist in the urothelium, rather than being restricted solely to renal parenchymal compartments [118]. Others have detected BKPyV in non-renal tissues such as the CNS [119] or the lungs [120], very rarely under extreme immunosuppression. Whether these represent true additional reservoirs or simply an endpoint of disseminated reactivation remains unresolved.

JCPyV similarly persists in the kidney, but increasing evidence suggests a multi-compartment persistent profile. The archetype strain of JCPyV establishes a chronic, asymptomatic infection in the urinary tract that can last for life for most individuals [2]. Additionally, JCPyV can be found at low levels in lymphoid and hematopoietic tissues of healthy carriers [121], raising the possibility that the virus latently “hides” in the immune cell compartment. A major unanswered question is whether JCPyV also resides in the CNS during latency. Some studies have reported JCPyV sequences in the brains of individuals without PML [122,123], suggesting that glial progenitor cells, choroid plexus epithelial cells, brain vascular pericytes, or leptomeningeal cells could serve as a “silent” reservoir [124,125] (Figure 1). In summary, it remains an open question whether BKPyV and JCPyV latency is maintained in a single favored cell type or spans multiple cell lineages with viral trafficking between compartments. Understanding how these viruses persist and occasionally reactivate after years of quiescence is essential.

### 4.2. Mechanistic Understanding of Persistence

Both BKPyV and JCPyV genomes persist as circular episomes in the host cell nucleus upon infection, where they are wrapped in host histones to form minichromosomes [45,117]. The architecture of viral chromatin, including nucleosome positioning and histone modifications, has been shown to be involved in maintaining viral latency. For JCPyV, latent genomes in cell culture can be reactivated by histone deacetylase inhibitors such as trichostatin A and sodium butyrate [126,127], indicating that the histone modification transcriptionally represses viral DNA expression. Consistently, JCPyV DNA in non-PML tissues is transcriptionally silent, which is believed to be likely associated with deacetylated histones [121]. For BKPyV, it is suspected to have a similar epigenetic silencing, but the chromatin-state details still remain unknown in vivo. Both viruses contain the NCCR, a critical regulatory element that undergoes rearrangement during persistence [2], where the archetype forms of the virus have an intact NCCR that restricts lytic gene expression [128], while rearranged NCCRs are often observed upon reactivation, leading to robust transcription and replication [129,130]. Then the open question is how chromatin structure might influence or be influenced by the NCCR rearrangements. Emerging high-resolution approaches, including single-cell and locus-resolved epigenomic methods, may now allow this longstanding question to be addressed directly by linking viral chromatin organization to transcriptional activity and replication at the level of individual viral episomes. A mechanistic understanding of latency at the chromatin level is still lacking, including what and how histone marks keep the viral minichromosomes quiescent and their stability.

Reactivation of BKPyV and JCPyV from the persistent state clearly correlates with changes in the physiological environment of the host, but the precise triggers remain poorly defined. Among all, impaired immune surveillance is strongly correlated with reactivation [2,55]. In immunosuppressed settings, such as AIDS, treatment with immunomodulatory therapies, and kidney transplantation, reduced virus-specific T-cell responses enable viral escape from the persistent state, resulting in active replication. Profound immunosuppression plays a pivotal role in driving the “selection” for more aggressive viral variants, such as the acquisition of NCCR rearrangements by JCPyV in AIDS patients or in patients treated with natalizumab, which converts the virus into a high-replication, neurotropic form [131,132]. Additionally, tissue microenvironmental cues are thought to modulate persistence. Pro-inflammatory signals such as TNF-α and IL-1β paradoxically activate the JCPyV promoter via the NF-κB pathway in vitro [121,127,133], suggesting that local inflammation or cell stress in the infected tissue could induce lytic, linking microenvironment changes to reactivation. Moreover, the host cell’s cellular differentiation or cell cycle state is likely to be importantly involved since polyomavirus LTAg can drive quiescent cells into S-phase to license viral DNA replication [96,134]. One hypothesis is that latency is favored in cells that are cell-cycle arrested or terminally differentiated, whereas a shift toward proliferation or a change in the host cell’s metabolic state might enable reactivation [135]. Notably, BKPyV reactivation has been observed during rapid cellular turnover or injury in the kidney [55,136]. Unfortunately, firm experimental evidence is lacking for pinpointing a particular cell cycle phase or metabolic trigger for reactivation. Further studies are required to determine whether entering a certain cell cycle or specific metabolic shifts can cause viral reactivation in latently infected cells.

The virus–host equilibrium for polyomaviruses is strikingly stable, as they can coexist with the host for decades without causing disease under usual conditions. As mentioned above, the equilibrium is actually maintained by a combination of viral latency mechanisms and continuous immune surveillance [121]. On the viral side, both BKPyV and JCPyV have evolved strategies to actively limit viral replication through viral miRNAs or regulatory proteins, such as agnoprotein and STAg, while avoiding elimination of susceptible host cells [35,109,137,138]. On the host side, the immune system continuously patrols and curtails polyomavirus activity through robust virus-specific T-cell responses, as evidenced by the sudden viral outbursts in conditions of compromised T-cell function [16,53,139,140]. The virus is never fully cleared but held at bay for exposed individuals, of which the mechanism is only partially understood. The virus restricts its own expression to maintain equilibrium, allowing the immune system to tolerate low-level viral presence without triggering immunity. It is not fully understood how latently infected cells survive under immune scrutiny over decades, possibly through active modulation of immune checkpoints. This virus–host stalemate remains a remarkable balance, where BKPyV and JCPyV effectively establish a chronic infection that neither replicates unchecked nor is eradicated. Such a fine-tuned balance is easily disturbed by any perturbation, which then results in severe clinical cases of PML or BK-nephropathy. Thus, unraveling these maintenance mechanisms will be crucial to devising interventions that prevent reactivation without forcing viral evolution or immune escape.

### 4.3. Transmission Routes and Life Cycle Ecology

Both BKPyV and JCPyV are ubiquitous viruses acquired early in life, but the exact modes of person-to-person transmission remain incompletely defined. A predominant view is that transmission occurs via contact with infected body fluids, especially urine. Infected individuals can shed viruses in their urine, exposing others through contact with hands, food, or water. Fecal-oral spread may also contribute, since polyomavirus DNA has been found in sewage and stool, indicating that ingestion of contaminated water or food could be a route [2,117]. Many seroprevalence studies have revealed that primary infection usually occurs in early childhood [141,142,143], implying that casual interpersonal contact within families or communities during childhood is a critical period for transmission. An alternative view is that the respiratory route could be an additional route for viral transmission. There are studies that detected JCPyV DNA in tonsillar tissues [40,57,144], suggesting inhalation of aerosolized virus or upper-respiratory tract exposure as an alternative infection route. BKPyV has also been detected in occasional cases of respiratory disease, such as causing pneumonia in immunocompromised hosts [55,120]. Although attempts to recover infectious JCPyV from saliva or nasopharyngeal swabs of known carriers have shown mostly negative results, BKPyV has been found in salivary gland tissues [145,146]. Even if the respiratory spread is a possible route, it is likely the least common pathway. Urine-oral contact remains the most substantiated route, but further epidemiological studies are needed to determine if additional pathways contribute measurably to polyomavirus transmission.

The shedding of both BKPyV and JCPyV from exposed individuals is a common way to create opportunities for transmission throughout their life cycle. In immunocompetent hosts, viral shedding is typically subclinical and sporadic. In healthy individuals, there is asymptomatic viruria occurring with low viral loads and appearing suddenly and without consequence. Longitudinal urine sampling shows BKPyV viruria can be particularly labile—exhibiting pronounced day-to-day variability and intermittency—whereas JCPyV can appear more persistent in some individuals, supporting the idea that urinary shedding “stability” differs by virus and host [147,148]. Host factors are found to be able to dramatically change the shedding pattern. Immunosuppression is the main accelerator of viral reactivation, leading to high-titer shedding, as illustrated by kidney transplant recipients, up to 80% of whom develop viruria post-transplant, and a significant fraction develop viremia, as the virus escapes local control [21,55,149]. JCPyV shows a similar pattern with the elevated viral loads in blood and urine under immunomodulatory conditions, presumably increasing shedding and transmission risk. Aside from iatrogenic immunosuppression, certain life stages and physiological conditions likely influence polyomavirus shedding. Surprisingly, pregnancy has been observed to be associated with increased BKPyV reactivation and shedding [150]. The relative immunosuppressive state of pregnancy and hormonal changes in women may transiently tilt the host–virus balance in favor of the virus. More generally, any condition that alters immune vigilance or causes cellular stress could affect shedding dynamics, which needs further evidence.

## 5. Conclusions

Clarifying transmission routes and shedding determinants has practical implications. If respiratory or other non-urinary routes prove significant, infection control guidelines may need to be broadened. Understanding the life-cycle ecology, including when infections occur and who is affected, could also inform strategies such as the timing of vaccination. As it stands, the high prevalence of BKPyV and JCPyV worldwide and their usually benign nature suggest a long-standing coevolution with humans, with transmission entwined in our early development and communal living. Persistence, not pathogenesis, is the primary evolutionary strategy of BK and JC polyomaviruses. Understanding the persistence mechanism is essential for the clinical management of reactivation diseases, such as PML and BK-PVAN.

Unraveling the fine points of this transmission will complete our understanding of the polyomavirus life cycle and help predict or prevent rare cases when these ubiquitous “innocent” viruses become pathogenic. Filling the knowledge gaps about reservoirs, molecular regulation, and transmission will reshape how we conceptualize human polyomavirus biology.

## Data Availability

No new data were created or analyzed in this study.
